# Well-Being and Cognitive Factors Influencing Health Care Workers’ Adherence to Internet-Based Stress Management: Mixed Methods Analysis of a Nonrandomized Controlled Study

**DOI:** 10.2196/73306

**Published:** 2026-04-02

**Authors:** Miqi Li, Quan Wang, Danli Zheng, Danni Feng, Xiaorong Lang

**Affiliations:** 1 Department of Nursing, Tongji Hospital, Tongji Medical College, Huazhong University of Science and Technology, No.1965 Jiefang Road, Wuhan, Hubei, 430030, China, 86 13986214079; 2 Nursing School, Tong Medical College, Huazhong University of Science and Technology, Wuhan, Hubei, China

**Keywords:** adherence, stress management, internet-based intervention, fatigue, work-related stress, health care workers, user experience

## Abstract

**Background:**

High stress levels are common among health care workers (HCWs), threatening their health and workforce stability. Internet-based mobile stress management (MSM) is a promising intervention for reducing work-related stress; however, poor adherence limits effectiveness. Exploring factors influencing HCWs’ adherence may thus aid in developing optimal interventions.

**Objective:**

The research aimed to investigate (1) how HCWs’ well-being and cognitive factors influenced MSM treatment adherence and (2) what HCWs’ specific needs for MSM were.

**Methods:**

This study was a convergent mixed methods secondary analysis of a nonrandomized controlled trial. HCWs who were currently employed, had internet access, had no serious medical problems, and were willing to participate were recruited by convenience sampling through an MSM project in a large Chinese general hospital from August 11, 2021, to January 31, 2022. Those intending to leave the hospital or with insufficient medical condition for follow-up were excluded. Quantitative data were collected from 157 HCWs (n=135, 86% female participants; mean age of 33.7, SD 4.9 y) electronically via Research Electronic Data Capture (REDCap). Measures included sociodemographic characteristics, the Fatigue Assessment Scale, the 14-item Perceived Stress Scale, a user experience questionnaire, an attitudes scale (perceived usefulness, feasibility, and enjoyment), and self-reported practice frequency. Qualitative data were collected via an open-ended question answered by 96 participants. Quantitative data were analyzed using hierarchical regression and structural equation modeling. Qualitative data were analyzed using reflexive-thematic analysis in NVivo (QSR International).

**Results:**

In the quantitative study (n=157), hierarchical regression analyses showed that fatigue was a significant negative predictor of adherence (b=−0.050, 95% CI −0.086 to −0.015; *t*_149_=−2.859; 2-tailed *P*=.005), while user experience (b=0.074, 95% CI 0.042-0.106; *t*_148_=4.569; 2-tailed *P*<.001) and attitudes (b=0.238, 95% CI 0.140-0.336; *t*_147_=4.863; 2-tailed *P*<.001) were positive predictors. Results from the structural equation model demonstrated a significant indirect effect of user experience on adherence through attitudes (indirect effect=0.047, 95% CI 0.021-0.083; *P*<.001), but no significant direct effect. Separately, qualitative data of responses (n=96) identified four themes about the experience, namely, (1) individualized intervention, (2) effective feedback, (3) reward and constraint mechanisms, and (4) duration of intervention.

**Conclusions:**

This mixed methods study innovatively integrates quantitative and qualitative needs assessment to elucidate the adherence pathway in digital stress management for HCWs. It differentiates itself from prior research by moving beyond merely describing adherence barriers by quantifying the mediating role of attitudes between user experience and treatment adherence. The study indicates that despite fatigue barriers, improving user experience enhances positive attitudes toward MSM, which is vital for ensuring treatment adherence. This contributes to the field by elucidating a key psychological pathway for engagement. For real-world implications, to optimize user experience, future studies should consider focusing on individualized needs, providing effective feedback, developing reward and constraint mechanisms, and designing flexible intervention durations.

## Introduction

Health care workers (HCWs) are often more vulnerable to work-related stress due to multiple stressors that exist in health care contexts, including long working hours, excessive workload, shift work, and inadequate staffing [1-3]. Moreover, the postpandemic era may require HCWs to spend more energy and time on extra work, which places them in a more strained and stressful situation [4,5]. Data released by the World Health Organization in 2025 revealed that one-third of physicians and nurses experience depression or anxiety, with over 10% having experienced suicidal or self-harm ideation [6]. In line with this global pattern, a recent nationwide study among Chinese medical professionals revealed that 28.2% and 24% of participants exhibited major depressive and anxiety symptoms, respectively, with the accumulation of multiple work-related stressors significantly elevating mental health risks [7]. In general, it estimates indicate that the prevalence of stress among HCWs ranges from 29.8% to 63% [8]. The detrimental effects of overwhelming work-related stress on HCWs have been well established, such as mental disorders, sleep disturbance, and physical symptoms [9-11]. Long-term work-related stress may also reduce patient satisfaction and jeopardize patient safety [12,13]. Hence, implementation of stress management interventions is essential in improving the personal health of HCWs and further ensuring the stability of health care human resources.

For now, several stress management techniques, including mindfulness, deep breathing, and regular exercise, have been identified as simple to apply and effective in alleviating both the physical and psychological indices of stress [14-16]. However, adherence to stress management behaviors may not be that easy for HCWs with shift work and irregular working schedules. A recent mixed methods study concluded that cumulative stressors would hinder the application of recommended stress management techniques, resulting in half of them failing to adhere to stress management behaviors [17]. All these findings suggest that it is critical to develop an adaptable and accessible stress management intervention so that HCWs can better use stress management strategies to cope with daily stress.

With the widespread use of digital devices, accumulative research evidence has suggested that mobile stress management (MSM) intervention may produce promising effects on improving stress symptoms and well-being in the workplace [18-20]. However, unlike traditional interventions in which the psychotherapist may supervise the adherence to treatment through face-to-face communications, the adherence of online participants has always been a bottleneck issue in such interventions [21,22]. Recent studies have suggested that personal support might enhance the adherence to internet-based interventions rather than a “pure” self-guided intervention, and the effects varied with the intensity of the intervention [23]. For example, a randomized study found that the adherence rate for stress management in the content-focused guidance sample was 70.5%, higher than both the adherence-focused guidance sample (68.8%) and the administrative guidance sample (42%) [20]. On the one hand, personal guidance may be regarded as an effective approach to improving adherence to internet-based stress management; on the other hand, it would be meaningful to find out factors that influence interindividual differences in nonadherence to such interventions.

Although personal guidance can improve adherence to internet-based stress management, understanding the individual factors underlying nonadherence remains critical. Notably, significant gaps persist regarding how these factors function specifically in MSM interventions targeting HCWs. Previous research identified that well-being might be a critical individual factor that might influence treatment adherence. For example, a qualitative study conducted among general populations identified that high stress levels and fatigue symptoms would hinder individuals from prioritizing and engaging in internet-based interventions [24]. However, another study conducted among HCWs suggested that high workload rather than stress levels may act as a barrier to web-based interventions for modifying their lifestyle, including physical activity [25]. This might be explained by the fact that the appraisal of stress can vary among various people, while some individuals presume stress as a “hindrance,” others would identify it as a “challenge” instead [26]. Consequently, the extent to which high stress levels act as a hindrance for health care professionals is not very definite. It would be fascinating to investigate how individual well-being, as expressed by stress and fatigue complaints, impacts HCWs’ adherence to MSM.

Meanwhile, cognitive elements of therapy adherence should be considered. Users’ perspective on these intervention components or user experience cannot be overlooked. However, research specifically examining how user experience influences adherence to MSM among HCWs remains limited. Because work-related stress can cause a wide range of symptoms, including psychological, physical, and behavioral problems [27-29], those who experience different stress symptoms will require different interventions [30]. According to the Technology Acceptance Model models, individuals will only implement and use an intervention if they believe it is valuable and feasible [31]. To build an effective tailored stress management intervention, it would be required to focus on the user experience and investigate its effects on attitudes and adherence to MSM. This represents a significant gap in the current literature, particularly regarding HCW populations.

To summarize, the current research sought to investigate (1) how HCWs’ well-being and cognitive factors influenced their treatment adherence to MSM and (2) what HCWs’ requests for MSM were. In particular, we suggested the following research hypotheses:

 *H*1*:* Individual well-being has a significant influence on HCWs’ treatment adherence, and fatigue has a negative effect, although stress levels do not.

 *H*2*:* The user experience of HCWs would have significant effects on their perceived feasibility and use.

 *H*3*:* The attitudes would have significant effects on HCWs’ treatment adherence.

 *H*4*:* The attitudes of HCWs would significantly mediate the impact of their user experience on treatment adherence.

## Methods

### Study Design

This study was a convergent mixed methods secondary analysis of a nonrandomized controlled trial [32,33]. It aimed to investigate the influence of HCWs’ well-being and cognitive variables on their adherence to MSM use. This study was reported in compliance with the Good Reporting of a Mixed Methods Study [34]. We acquired secondary data from HCWs who participated in the MSM program at a large general hospital in China from August 11, 2021, to January 31, 2022 [32,33]. Information on the research intervention can be found on the ClinicalTrials.gov website (NCT05239065).

### Inclusion and Exclusion

The inclusion criteria were (1) currently employed, (2) self-reported having internet access, (3) did not have any serious medical problems (eg, cancer or stroke), and (4) were willing to participate in this study and sign the consent form. Participants were excluded if they (1) intended to leave the hospital or units for any reason or (2) were in insufficient medical condition to be followed up on.

### Participants Characteristics

Sociodemographic characteristics included sex (male or female), age (y), marriage status (married or unmarried), educational background (undergraduate degree or postgraduate degree), position (physician, nurse, or other), working hours (h), income (Chinese Yuan [CNY]), and BMI.

### Sampling Procedures

A convenience sample of HCWs was recruited from a large general hospital in China between August 11, 2021, and January 31, 2022. Participants were enrolled from an ongoing MSM program.

### Sample Size, Power, and Precision

This study was a secondary analysis of the original MSM program data. In the primary trial, 179 participants were ultimately allocated to and received the intervention. For the purposes of the current analysis, which designated the Fatigue Assessment Scale (FAS) as a key outcome, we applied a strict criterion of complete data: any record with one or more missing items on the 10-item FAS was excluded to ensure the integrity of the primary outcome measure without imputation. According to the inclusion and exclusion criteria, after applying this data completeness rule, 157 participants were ultimately enrolled and completed the quantitative survey. The sample size for the qualitative component was guided by the principle of information saturation, whereby recruitment continued until no substantially new themes emerged from the data. Ultimately, 96 individuals voluntarily agreed to participate.

### Measures and Covariates

#### Well-Being Factors

Fatigue symptoms were measured using the FAS, a regularly used tiredness measure consisting of 10 questions, was used to assess fatigue symptoms [35]. Each item was graded using a 5-point Likert scale ranging from 0 (never) to 4 (always). Those with a higher FAS total score had more severe fatigue symptoms. The FAS has a Cronbach α of 0.883, suggesting that the internal reliability of this scale was satisfactory in this study.

Perceived stress was assessed using the 14-item Perceived Stress Scale (PSS-14) to gage stress levels at the baseline. PSS-14 is an internationally used self-reported 5-point Likert scale, with a greater total score indicating a higher degree of stress [36]. The Cronbach α was 0.835, indicating that the PSS-14 was a credible instrument in this study for measuring felt stress.

#### Cognitive Factors

User experience was evaluated using an 8-item questionnaire (“Can you tell us about your experience with this research? [in terms of studying organization, quality of staff for communication or explanation, quality of videos, quality of instructional brochure, quality of education regarding the effects of stress on health, quality of education regarding stress management strategies, quality of weekly feedback reports, and portable device and app applications]”) with a 5-point Likert scale (1 [extremely terrible] to 5 [extremely excellent]), and the overall user experience score varied from 8 to 40, with a higher score indicating a better user experience. The Cronbach α coefficient for these 8 items was 0.933, indicating that the 8-item questionnaire was trustworthy for assessing HCW user experience.

Attitudes were assessed on three dimensions: perceived use, perceived feasibility, and perceived enjoyment. Perceived usefulness was defined as “the level of one’s belief in using technology,” perceived feasibility was defined as “a person’s belief in the use of a technology that can be easily used,” and perceived enjoyment was defined as “the degree of enjoyment of using a technology,” as suggested by the previous study. We specifically investigated HCW sentiments using three items: (1) “Did you find the stress management intervention study beneficial in general?” (perceived use), (2) “Did you find the stress management intervention study to be straightforward to complete?” (feasibility), and (3) “Did you find the stress management intervention study interesting overall?” (perceived enjoyment). The 3 items were graded on a 5-point Likert scale, with higher scores suggesting a more favorable attitude to MSM. The Cronbach α coefficient for these 3 items was 0.713, showing that the 3-item questionnaire was consistent enough to assess attitudes against MSM among HCWs.

#### Treatment Adherence

Treatment adherence was measured by self-reported practice frequency of three stress management techniques: physical activity, deep breathing exercises, and mindfulness, using a 7-point Likert scale (“Did you practice the stress management interventions?” 1 [never] to 7 [everyday]). A higher overall score suggested more MSM adherence. The Cronbach α coefficient for these 3 items was 0.793, indicating that the 3-item questionnaire was trustworthy in assessing HCWs’ adherence to MSM.

#### Open-Ended Survey

In order to learn about the unique needs of HCWs when using MSM, we requested HCWs to answer an open-ended question (online interview), “Do you have any suggestions for improving the mobile stress management program?”

#### Data Collection

Research Electronic Data Capture (REDCap), a web-based online survey tool, was used to capture both quantitative and qualitative data. The MSM was a human-assisted intervention that provided weekly feedback and online stress management education. Stress management training was provided by a neuropsychologist in the form of video clips, posters, and messaging. Participants were encouraged to use at least 1 of the 3 recommended stress management approaches on a weekly basis (physical activity, deep breathing, and mindfulness). Participants were asked to keep a weekly journal that documented their practice frequency and mood state, and they were subsequently given feedback based on their replies. Details of the intervention and data collection have been published previously [32,33].

### Statistical Analysis

#### Overview

This study used a convergent mixed methods design. Qualitative and quantitative data were collected concurrently but analyzed separately. The integration occurred during the interpretation phase through triangulation; the qualitative findings (user-identified needs and preferences) were used to explain and contextualize the quantitative results (statistical predictors of adherence). This approach allowed us to understand not only what factors influence adherence but also why and how they matter from the users’ perspective.

#### Quantitative Data Analysis

IBM SPSS (version 26.0; IBM Corp) and IBM SPSS Amos (version 28.0; IBM Corp) were used to analyze quantitative data. For each categorical item, a descriptive analysis of the response frequencies was performed, and the mean (SD) for the continuous variables was calculated. Then, hierarchical regression analyses were run to examine the relative contributions of demographic variables, well-being factors, and cognitive factors in treatment adherence variances. Four sequential models were tested, with *R*^2^ change indicating the additional variance explained at each step and a 2-side α level of .05.

To test the hypothesized mediation model, structural equation modeling with maximum likelihood estimation was used. The mediation effect of attitudes between user experience and adherence was examined using bootstrapping with 5000 resamples. Model fit was assessed using multiple indices including the *χ*²/df ratio (3), root mean square error of approximation (<0.08), standardized root mean square residual (<0.08), comparative fit index (>0.90), and Tucker-Lewis index (>0.90) [17]. The indirect effects were examined, and a significant effect was determined if the 95% CI did not include zero.

#### Qualitative Data Analysis

QSR NVivo 14.0 was used to analyze qualitative data. The open-ended survey responses were analyzed using a pragmatic approach to reflexive-thematic analysis [37]. The analysis was carried out in six steps: (1) familiarization with the data, (2) code generation, (3) theme construction, (4) review of potential themes, (5) theme definition and naming, and (6) report production. In this stage of the study, 2 experienced researchers (ML and QW) independently read the transcript and coded the findings. Both researchers then met to compare their coding and discuss any discrepancies until a consensus was reached on the thematic structure.

### Ethical Considerations

This study was approved by the Institutional Review Board (IRB) of the Medical Ethics Committee of Tongji Medical College, Huazhong University of Science and Technology, China (approval number: IRB number 2021S141). All participants provided informed consent electronically prior to participation. For the secondary analysis reported in this manuscript, the original IRB approval and consent procedures permitted the use of deidentified data. All data were collected and stored anonymously using coded identifiers. No personal identifying information was linked to the survey responses, ensuring participant confidentiality. Participants did not receive any financial compensation for their involvement in the study. This manuscript does not contain any personal images, audio or video recordings, or text excerpts that could lead to the identification of an individual participant.

## Results

### Participant Characteristics

There were 157 participants in total, with 135 (86%) female participants and 22 (14%) male participants. In terms of age, the mean age was 33.7 (SD 4.9) years, 35 (22.3%) participants were under the age of 30 years, 97 (61.8%) were between the ages of 30 and 40 years, and 25 (15.9%) were over the age of 40 years. In terms of marital status, 115 (73.2%) participants were married, while 42 (26.8%) were single. In terms of position, 30 participants (19.1%) were physicians, 104 (66.2%) were nurses, and 23 (14.7%) were other HCWs. When it came to family income, 18 (11.5%) of the participants had less than 10,000 CNY (1 CNY≈0.155 USD). 81 (51.6%) participants had a family income of 10,000 to 15,000 CNY, 35 (22.3%) had a family income of 15,001 to 20,000 CNY, and 23 (14.6%) had a family income of more than 20,000 CNY. When it came to working hours, 22 (14%) participants worked less than 35 hours per week, 74 (47.1%) worked 35 to 44 hours per week, and 61 (38.9%) worked more than 44 hours per week (Table 1).

**Table 1.** Baseline characteristics of participants in the quantitative component of a mixed methods study on mobile stress management adherence (n=157)^.^

Characteristics	Participants, n (%)
Sex	
Female	135 (86)
Male	22 (14)
Age group (y)	
<30	35 (22.3)
30 to 40	97 (61.8)
>40	25 (15.9)
Marital status	
Married	115 (73.2)
Unmarried	42 (26.8)
Educational level	
Undergraduate degree	120 (76.4)
Postgraduate degree	37 (23.6)
Occupation	
Physician	30 (19.1)
Nurse	104 (66.2)
Other health care workers	23 (14.7)
Income (Chinese Yuan)^a^	
<10,000	18 (11.5)
10,000 to 15,000	81 (51.6)
15,001 to 20,000	35 (22.3)
>20,000	23 (14.6)
Working (h)	
<35	22 (14)
35 to 44	74 (47.1)
>44	61 (38.9)
BMI	
≤23.9	120 (76.4)
>23.9	37 (23.6)

a Income is reported in Chinese Yuan; the average exchange rate was 1 Chinese Yuan≈0.155 USD during the study period of 2021‐2022.

### Descriptive Statistics

Table 2 displays descriptive results for the main study variables. Participants rated perceived usefulness as 2.96 out of 5.0 (SD 0.85), perceived feasibility as 3.71 out of 5.0 (SD 0.72), and perceived enjoyment as 3.59 out of 5.0 (SD 0.69), in that order. The HCWs rated their experience with MSM as 32.03 out of 40.0 (SD 5.01), and the “quality of weekly feedback reports” received the highest mean score of 4.17 (SD 0.73). Participants rated their adherence to MSM for physical activity as 2.91 out of 5.0 (SD 1.15), deep breathing as 3.25 out of 5.0 (SD 1.34), and mindfulness as 2.92 out of 5.0 (SD 1.33), on average.

**Table 2.** Descriptive statistics of attitude, use experience, and adherence to mobile stress management in quantitative component (n=157).

Variables	Mean (SD; range)
Experience^a^	32.03 (5.01; 8-40)
Experience regarding study organization	3.74 (0.71; 1-5)
Quality of staff for communication or explanation	4.08 (0.77; 1-5)
Quality of the videos	4.03 (0.80; 1-5)
Quality of education regarding the effects of stress on health	4.09 (0.77; 1-5)
Quality of education regarding stress management strategies	4.06 (0.77; 1-5)
Quality of weekly feedback reports	4.17 (0.73; 1-5)
Applications of portable device and app	4.01 (0.75; 1-5)
Attitude^a^	10.26 (1.82; 3-15)
Perceived usefulness	2.96 (0.85; 1-5)
Perceived feasibility	3.71 (0.72; 1-5)
Perceived interest	3.59 (0.69; 1-5)
Adherence to mobile stress management^a^	9.15 (3.22; 3-21)
Physical activity	2.97 (1.15; 1-7)
Deep breathing	3.25 (1.34; 1-7)
Mindfulness	2.92 (1.33; 1-7)

a Composite score of questionnaire.

### Hierarchical Regression Analyses

For the hierarchical regression analysis, 4 models were obtained. As shown in Table 3, the demographic variables (sex, age, marital status, position, income, and working hours) were entered into the model in the first step, and no variable was found to be significant in explaining the variance of treatment adherence. When perceived stress and fatigue symptoms were included in step 2, the percentage of treatment adherence explained by the predictors significantly increased from *R*²=0.043 in model 1 to *R*²=0.093 in model 2, and fatigue symptom was demonstrated to be a significant predictor (*b*=−0.050; *t*_149_=−2.859; and 2-tailed *P*=.005). Then, after user experience was included as the predictor in step 3, the percentage of treatment adherence explained by the predictors significantly increased from *R*²=0.093 in model 2 to *R*²=0.205 in model 3, and user experience was demonstrated to be a significant predictor (*b*=0.074*; t*_148_=4.569; and 2-tailed *P*<.001). In the last step, when attitude was included as the predictor, the percentage of treatment adherence explained by the predictors significantly increased from *R*²=0.205 in model 3 to *R*²=0.315 in model 4, while attitude was shown to be a significant predictor (*b*=0.238; *t*_147_=4.863; and 2-tailed *P*<.001), the significance of user experience was shown to be disappearing in model 4 (*b*=0.031; *t*_147_=1.770; and *P*=.08). All the predictors of model 4 together explained 31.5% of the variance of treatment adherence (*R*²=0.315; *P*<.001).

**Table 3.** Hierarchical regression analysis of mobile stress management adherence (n=157).

Predictors	Model 1 (*R*²=0.043; *F* test (5, 151)=1.385)			Model 2 (*R*²=0.093; *F* test (7, 149)=2.182^a^)			Model 3 (*R*²=0.205; *F* test (8, 148)=4.774^b^)			Model 4 (*R*²=0.315; *F* test (9, 147)=7.520^b^)		
	*b* ^c^	β^d^	2-tailed *t* test (151)	*b*	β	2-tailed *t* test (149)	*b*	β	2-tailed *t* test (148)	*b*	β	2-tailed *t* test (147)
Constant	1.750	—^e^	3.773^b^	2.505	—^e^	3.789^b^	0.076	—^e^	0.093	−0.814	—^e^	−1.040
Sex	0.095	0.031	0.359	0.100	0.032	0.383	0.174	0.056	0.708	0.082	0.027	0.358
Age	0.185	0.084	0.997	0.170	0.078	0.934	0.078	0.036	0.452	−0.068	−0.031	−0.416
Position	0.113	0.131	0.865	0.051	0.035	0.389	0.002	0.001	0.014	0.033	0.023	0.292
Income	−0.185	−0.151	−1.785	−0.173	−0.141	−1.701	−0.119	−0.097	−1.241	−0.052	−0.042	−0.573
Working hours	0.151	0.069	0.794	0.167	0.076	0.883	0.192	0.088	1.084	0.174	0.079	1.054
Perceived stress	—	—	—	0.026	0.152	1.634	0.017	0.100	1.140	0.013	0.076	0.921
Fatigue symptoms	—	—	—	−0.050	−0.264	−2.859^f^	−0.041	−0.214	−2.452^f^	−0.038	−0.201	−2.468^f^
User experience	—	—	—	—	—	—	0.074	0.345	4.569^b^	0.031	0.144	1.770
Attitudes	—	—	—	—	—	—	—	—	—	0.238	0.402	4.863^b^

a *P*<0.05.

b *P*<0.001.

c *b*=unstandardized coefficient.

d β=standardized coefficient.

e Not applicable.

f *P*<.01.

### Mediation Analysis

According to Hu and Bentler’s [38] suggested model fit indicators, the model in this paper had a good fitting degree. Table 4 shows that the *χ²*/df values were less than 3, the root mean square error of approximation and standardized root mean square residual values were less than 0.80, and the nonnormed fit index, comparative fit index, and adjusted goodness of fit index values were all greater than 0.90, all of which were within the range of acceptable fit indexes. To summarize, the results of confirmatory factor analysis confirm that the empirical model comprising attitude, experience, and adherence has good validity; the collected data are highly reliable; and the research findings are robust.

**Table 4.** Goodness of fit indices for the structural equation modeling testing the mediation effect of attitudes.

Index	Goodness-of-fit measure		Model fit
	Good fit	Accept fit	
*χ²*/*df*	0≤ *χ²*/*df* ≤2	2 <*χ²*/*df* ≤3	1.640
*P* value	0.05< *P* ≤1.00	0.01< *P* ≤0.05	0.073
RMSEA^a^	0≤ RMSEA ≤0.05	0.05< RMSEA ≤0.08	0.064
SRMR^b^	0≤ SRMR ≤0.05	0.05< SRMR ≤0.08	0.061
NNFI^c^	0.97< NNFI ≤1.00	0.95≤ NNFI ≤0.97	0.951
CFI^d^	0.97< CFI ≤1.00	0.95≤ CFI ≤0.97	0.980
AGFI^e^	0.90< AGFI ≤1.00	0.95≤ AGFI ≤0.97	0.928

a RMSEA: root mean square error of approximation.

b SRMR: standardized root mean square residual.

c NNFI: nonnormed fit index.

d CFI: comparative fit index.

e AGFI: adjusted goodness of fit index.

**Figure 1.**
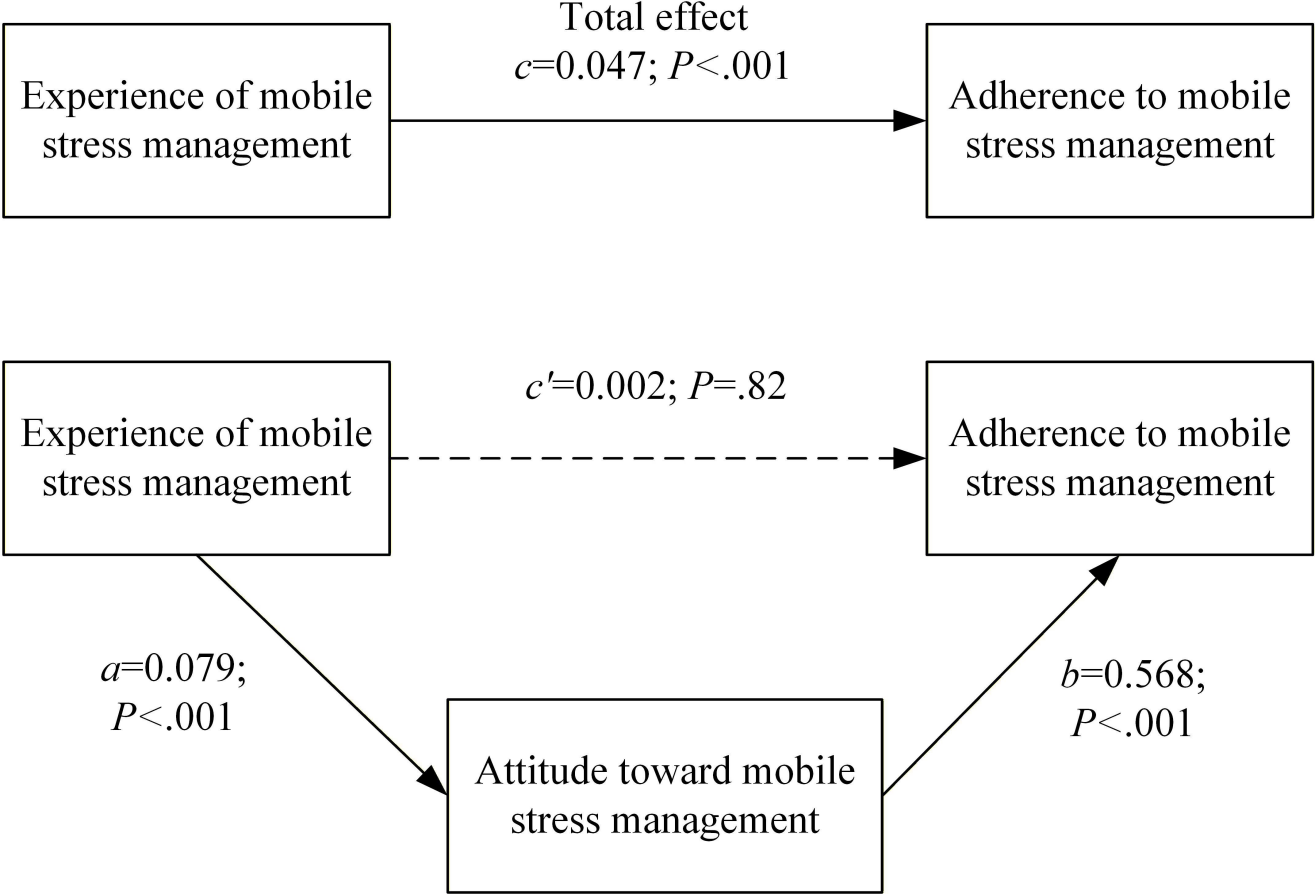
The mediating role of attitudes in the relationship between user experience and adherence to mobile stress management.

Figure 1 depicts the results of attitude mediation between experience and adherence to MSM. The path coefficient from experience to attitude was 0.079 (*P*<.001), and the path coefficient from attitude to adherence was 0.568 (*P*<.001). Meanwhile, the Bootstrapping estimates revealed that the direct influence of experience on adherence was not significant (direct effect=0.002; *P*=.86), implying that experience had a full indirect effect on adherence via the attitude toward MSM (indirect effect=0.047; *P*<.001).

### Results of Open-Ended Survey Response

The open-ended survey received 96 responses from 157 participants, which were identified and categorized into four themes: (1) individualized intervention, (2) effective feedback, (3) reward and constraint mechanisms, and (4) duration of intervention. Each theme could have 3 subthemes. Table 5 summarizes the themes, subthemes, and sample quotes.

**Table 5.** Themes, subthemes, and sample quotes from the open-ended survey responses regarding needs for improving mobile stress management in qualitative analyses (n=96).

Themes and subthemes	Sample quotes
Individualized intervention	
Diversified modules	*“Everyone’s stress sources are different and require one-on-one counseling from professionals.”* (Participant 79) *“I want to learn more about how to alleviate my anxiety.” *(Participant 118) *“I find it hard to change my sleep quality.” *(Participants 83 and 107) *“I want to know how to reduce negative emotions and deal with doctor-patient conflicts.” *(Participant 100)
Adapt to work schedules	*“I often work too long hours, and I find it difficult to guarantee enough time to practice those techniques.” *(Participant 9) *“I didn't have time to exercise.”* (Participant 26)
Staging the intervention	*“It’s hard to get started.”* (Participant 137) *“It’s hard to insist on.”* (Participant 14, 32, 50, 128) *“Professionals are required to follow up and guide the practice process.”* (Participant 79)
Effective feedback	
Feedback content	*“The content of the feedback should be more specific to the weekly plan and monthly schedule, which would be more intuitive.” *(Participant 68) *“The weekly questionnaire should be adapted to each individual’s situation.”* (Participant 41) *“It would be better if we could get summarized and analyzed feedback data on a weekly or biweekly basis.”* (Participant 147)
Feedback frequency	*“More frequent reminders are needed.”* (Participant 143) *“We need more supervision and feedback.”* (Participant 117)
Reward or constraint mechanisms	
Motivations for practice	*“Giving rewards to stimulate movement would be useful.” *(Participant 108) *“It would work by motivating everyone to exercise through group check-ins.”* (Participant 50) *“It’s important to mobilize the wisdom of everyone in the group and share more methods, skills, and experiences together.”* (Participant 156)
More restraints	*“It is completely dependent on self-consciousness and self-restraint.”* (Participant 158) *“It would be best if participants could be supervised and demanded a little more.”* (Participant 79) *“A little more restraint is needed to help us form habits.” *(Participant 96)
Duration of intervention	
Short-term intervention	*“Reduce the experimental cycle.”* (Participant 18)
Long-term intervention	*“I hope the project will continue for a longer time.”* (Participant 54, 90)

## Discussion

### Principal Findings

This research focused on how individual treatment adherence differed depending on their well-being and cognitive factors. By using a convergent mixed methods approach, we specifically examined the direct effects of well-being factors on treatment adherence and the indirect effects of cognitive factors on treatment adherence, as well as learning about the specific requirements of HCWs for MSM interventions.

Overall, adherence to the internet-based MSM was generally modest, with mindfulness and physical activity techniques showing lower adherence compared to deep breathing exercises. The results are consistent with previous studies [20,22], despite the reduction in dropouts in the internet-based intervention compared to face-to-face, participants often fail to have complete adherence. While physical activity and mindfulness are evidence-based techniques, their lower adherence in our sample may relate to the demanding work schedules of HCWs [8,16,19,39]. A previous study showed that breathing exercises can reduce stress levels even in the short term (mean time of 1.9, SD 1.2 min) [40], which means that breathing exercises may be more available for HCWs who spend most of their time providing health care. HCWs are a special group of people with heavy workloads and limited job autonomy. The time spent setting requirements and the accessibility of management programs were priorities in stress management for this group of people. Therefore, in the future, the stress management of specific interventions needs to be based on the working conditions such as the working hours or environment of different HCWs, taking into account the feasibility of developing a personalized management plan to improve adherence.

On the issue of burnout symptoms, this study revealed that fatigue symptoms were a significant predictor rather than perceived stress. An explanation for this finding is that perceived stress did not reach a statistically significant level in relation to burnout. One possible reason for this could be attributed to insufficient training hours, while another factor may be the nurses’ anticipation of encountering stressful work in their future shifts. Additionally, according to Maslow’s hierarchy of needs theory, fatigue symptoms represent fundamental and immediate needs, whereas perceived stress can potentially be managed through various methods by HCWs. As professional practitioners, HCWs consciously adapt themselves to reduce stress levels; however, it is important to consider potential biases resulting from psychological effects during the design phase of this study.

Our findings regarding the prominence of fatigue over perceived stress align with broader postpandemic research on psychological well-being, which suggests a shift in the salient components of well-being. Babazadeh [41], using a multidimensional measure of psychological well-being, found that factors like health locus of control and social support were significant predictors, underscoring the importance of targeting specific well-being components and cognitive factors in interventions rather than focusing solely on global stress measures. The significant role of fatigue in our study may reflect its centrality within the somatic symptoms dimension of well-being, which appears highly relevant for HCWs. Furthermore, fatigue and burnout may have intertwined effects, as evidenced by a prospective cohort study reporting that individuals in the “burnout and fatigue” group had a higher proportion of chronic course type compared to those experiencing “pure burnout” or “pure fatigue.” Severe burnout and fatigue symptoms become interconnected with each other as well as with other psychological and/or somatic complaints [42]. However, the specific association between fatigue and specific factors remains unclear [43].

The most interesting finding is that the attitude mediates experience and adherence. Our mediation analysis revealed a significant indirect effect of user experience on adherence through attitudes, while the direct effect was not significant. Most existing studies have emphasized the importance of user experience on treatment adherence [8,20]. However, the direct relationship between the experience and adherence is limited to a definite explanation. On the contrary, the discussion of attitude and adherence is a classic issue, and the amount of evidence reports the relationship between attitude and adherence in stress management interventions [8,44,45]. Our research indicates that the direct impact of user experience on treatment adherence disappeared after inputting the variable of attitudes. An implication of the further relationship is the possibility that the improvement of experience with the object of adherence increasing is to make the attitude of the internet-based mobile program or management positive, despite the weak significant association between the experience and attitude. This may provide another perspective on retention in stress management, and a similar relationship in other psychological health of HCWs should be developed in the future.

We also identify the 4 aspects of individualized intervention, effective feedback, reward or constraint mechanisms, and duration of intervention as certain factors to adherence to internet-based MSM. Generally, all the factors highlight the personalized service throughout the whole intervention. The high quality and individual needs of intervention contents are more vital; inadequately supported contents are a hindrance. Furthermore, HCWs, as a special population with a health care education background, face the problem of their personal situations, may not have time to integrate exercises into their daily lives, and may need more personal conversations with the eCoach during the staging and feedback stages. This is in line with previous observations [46-48]. In the reward or constraint mechanisms, HCWs required the increasing demands outwards, which addressed the role of the organization in the interventions. Proving reminders, encouraging each other, and using rewards as the external triggers were taken as effective measures to increase adherence in other evidence [49,50]. To the duration of intervention, extensive studies have pointed out that stress was reduced with the increasing duration of intervention [9,51,52]. Considering that health care often requires demanding physical and psychological efforts, HCWs often have little energy to implement stress management strategies on a daily basis. Therefore, future studies may need to be more focused on less exhausting interventions for those working in health care contexts.

### Limitations

Some limitations in the paper also need to be taken into consideration. First, the exploratory study investigated a small sample size with a short-term intervention. Second, the data collection of qualitative research conducted online could result in lacking information. Furthermore, the implication of this paper is that participants in qualitative research may have been positively biased in their support of the intervention because all the interviewees finished the intervention. It would be valuable in future work to extend the sample, focus on those who chose to drop out of the intervention, and explore the reasons for dropping out.

### Implications

The findings of this study highlight a central pathway for engagement in digital interventions and point to clear practical directions. By innovatively integrating quantitative modeling with qualitative assessment, this research moves beyond merely describing barriers to adherence. It thereby differentiates itself from prior work by quantitatively establishing the critical mediating role of participant attitudes between user experience and sustained use. This contributes a key insight to the field: successful engagement depends not just on the intervention itself, but on fostering positive attitudes through that experience. Consequently, the real-world implication is straightforward. To optimize adherence, MSM programs for HCWs must prioritize enhancing user experience via personalized content, actionable feedback, incentive mechanisms, and flexible duration, all essential for cultivating the positive attitudes needed for long-term use in high-demand settings.

### Conclusion

In summary, by innovatively integrating quantitative modeling with qualitative assessment, this study demonstrates that the value of user experience in stress management adherence to internet-based mobile intervention is fully mediated by participant attitudes, highlighting a key insight that differentiates our work from previous barrier-focused research. For HCWs, especially in high-demand settings, adherence is enhanced by interventions that are perceived as personalized, with actionable feedback, incentive mechanisms, and flexible duration. Future research should incorporate additional mediating variables through larger-scale and longer-term studies to further elucidate these mechanisms.

## Abbreviations

FAS: Fatigue Assessment Scale
HCW: health care worker
IRB: Institutional Review Board
MSM: mobile stress management
PSS-14: 14-item Perceived Stress Scale
REDCap: Research Electronic Data Capture
